# Expression and Function of Eicosanoid-Producing Cytochrome P450 Enzymes in Solid Tumors

**DOI:** 10.3389/fphar.2020.00828

**Published:** 2020-06-09

**Authors:** Eric A. Evangelista, Christi W. Cho, Theresa Aliwarga, Rheem A. Totah

**Affiliations:** ^1^Department of Pharmacy, School of Pharmacy, University of Washington, Seattle, WA, United States; ^2^Department of Medicinal Chemistry, School of Pharmacy, University of Washington, Seattle, WA, United States; ^3^Department of Pharmaceutics, School of Pharmacy, University of Washington, Seattle, WA, United States

**Keywords:** epoxygenases, hydroxylases, cytochrome P450, epoxyeicosatrienoic acids, hydroxyeicosatetraenoic acids, 20-HETE, tumor, angiogenesis

## Abstract

Oxylipins derived from the oxidation of polyunsaturated fatty acids (PUFAs) act as important paracrine and autocrine signaling molecules. A subclass of oxylipins, the eicosanoids, have a broad range of physiological outcomes in inflammation, the immune response, cardiovascular homeostasis, and cell growth regulation. Consequently, eicosanoids are implicated in the pathophysiology of various diseases, most notably cancer, where eicosanoid mediated signaling is involved in tumor development, progression, and angiogenesis. Cytochrome P450s (CYPs) are a superfamily of heme monooxygenases generally involved in the clearance of xenobiotics while a subset of isozymes oxidize PUFAs to eicosanoids. Several eicosanoid forming CYPs are overexpressed in tumors, elevating eicosanoid levels and suggesting a key function in tumorigenesis and progression of tumors in the lung, breast, prostate, and kidney. This review summarizes the current understanding of CYPs' involvement in solid tumor etiology and progression providing supporting public data for gene expression from The Cancer Genome Atlas.

## Introduction

**Scheme 1:**
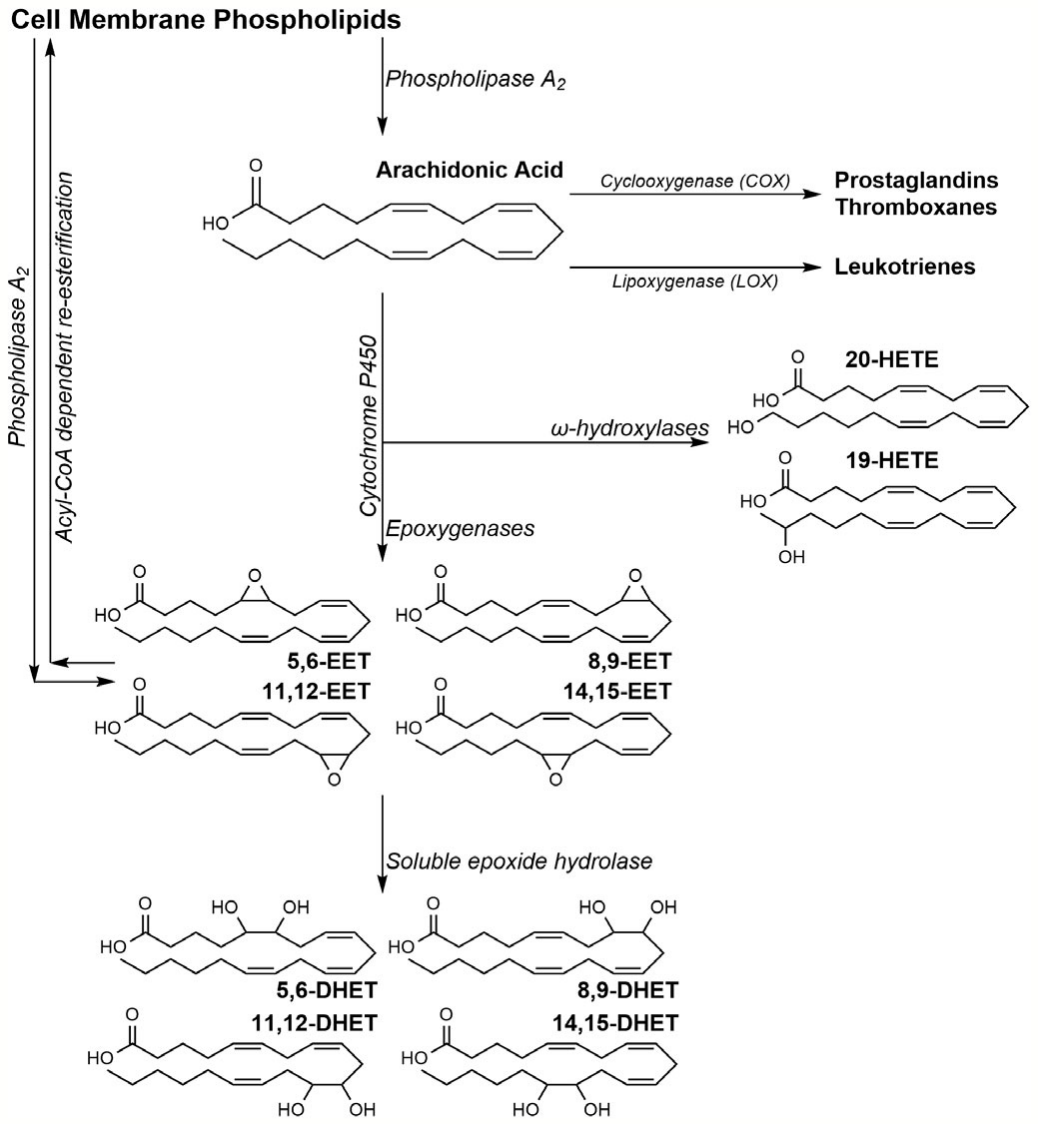
Arachidonic acid metabolism pathway.

Eicosanoids are a subset of physiologically active oxylipins derived from arachidonic acid (AA) oxidation. AA, esterified at the *sn2* position of lipid bilayer phospholipids, is released by phospholipase A_2_ (PLA_2_) in response to various cellular stimuli (Thomas et al., 1984). Upon release, AA can undergo oxidation *via* three pathways to produce multiple eicosanoids with distinct downstream effects (**Scheme 1**). The cyclooxygenase (COX) pathway results in prostaglandin H_2_, a precursor to prostacyclins, prostaglandins, and thromboxanes (Otto and Smith, 1995); the lipoxygenase (LOX) pathway generates leukotrienes and mid-chain AA-hydroxylated metabolite (Brash, 1999). AA metabolized by the third pathway, undergoes oxidation by cytochrome P450 (CYP) isoforms to two distinct classes of eicosanoids; the epoxyeicosatrienoic acids (EETs) and the hydroxyeicosatetraenoic acids (HETEs) (Capdevila et al., 1981; Proctor et al., 1987). Despite a common source, the numerous AA metabolites activate separate, sometimes overlapping, pathways to stimulate a broad array of physiological responses.

EETs are derived from the oxidation of AA's olefins by CYP epoxygenases to four possible regioisomers of *cis*-EETs (**Scheme 1**). EETs can stimulate a wide range of effects during inflammation, angiogenesis, and cardiovascular homeostasis (Node et al., 1999; Pozzi et al., 2005; Campbell and Fleming, 2010; Pfister et al., 2010). Due to their anti-inflammatory and pro-angiogenic activities, EETs have been investigated in recent years for their involvement in cancer progression and tumorigenesis (Spector et al., 2004; Panigrahy et al., 2011).

Another subset of CYPs, the hydroxylases, oxidize AA at various carbons to form many HETEs. (Powell et al., 1998; Choudhary et al., 2004). While CYP hydroxylases mostly prefer to oxidize AA at the terminal and penultimate carbons (ω and ω-1, respectively), metabolism on the mid-chain carbons has been observed. It is worth noting that some CYP isoforms function as both epoxygenases and hydroxylases generating a mixture of both EETs and HETEs (Choudhary et al., 2004).

CYP epoxygenases and hydroxylases are members of the larger CYP superfamily of membrane bound monooxygenases containing a heme prosthetic group in the active site (Ortiz de Montellano, 2005). Humans have 57 distinct CYP genes that encode for proteins and 46 pseudogenes (Ortiz de Montellano, 2005). Most research investigating changes in CYP expression during cancer progression is mainly focused on how these changes affect chemotherapeutic activation, clearance, or drug resistance. CYPs, specific to certain tumors, could potentially bioactivate agents as a novel targeted therapeutic approach, to concentrate the active, cytotoxic drug in tumor cells. Alternatively, CYPs could result in chemotherapeutic resistance due to their inherent ability to metabolize and often inactivate or expedite the clearance of xenobiotics thereby reducing their concentration in the tumor (Narjoz et al., 2014; van Eijk et al., 2019). CYP2J2, for example, is an isoform abundantly expressed in tumor tissues and metabolizes several antitumor tyrosine kinase inhibitors reducing their intracellular concentration (Narjoz et al., 2014). Similarly, CYP3A4 upregulation has been established in several breast cancer tumors and its expression levels are associated with docetaxel resistance (Miyoshi et al., 2002). Therefore, CYP expression in the context of cancer treatment is complex and CYP enzymes can act to enhance chemotherapeutic efficacy or diminish it.

This review compiles and summarizes the current status on the role of CYP epoxygenases and hydroxylases, and resulting EETs and HETEs, play in the development and progression of different sub-types of solid tumors. CYP gene expression data in tumor vs healthy tissue from The Cancer Genome Atlas (TCGA) database is analyzed and presented in figures to support findings. Where available, potential mechanisms and pathways involving CYPs, and their metabolites (**Table 1**), will be presented and novel chemotherapeutic strategies will be discussed for each tumor type.

**Table 1:** Summary of key CYPs in solid tumors.

CYP450 Isoform	Role in AA metabolism	Tumors where expression is observed	Increase/decrease expression and potential mechanism	References
CYP1A2	Epoxygenase	Lung cancer Bladder cancer	Unknown	(Rifkind et al., 1995)
CYP1B1	Epoxygenase/hydroxylase	Breast cancer Prostate cancer	Elevated enzyme levels -Increased EET/HETE production -Potential pro-angiogenic/pro-metastatic -Inhibits caspase-1 expression	(Finnström et al., 2001; Muskhelishvili et al., 2001; Chang et al., 2017)
CYP2C8	Major epoxygenase	Prostate cancer Renal cancer Bladder cancer	Potential increase in EET levels -promotes cell proliferation and survival -potential pro-angiogenic	(Sellmayer et al., 1991; Chen et al., 2001)
CYP2C9	Major epoxygenase	Lung cancer Prostate cancer Renal cancer Colorectal cancer Bladder cancer	Increased expression of CYP2C9, -increased EET levels -pro-inflammatory -pro-tumorigenic.	(Michaelis et al., 2003; Sausville et al., 2018; Wang et al., 2019)
CYP2C19	Epoxygenase	Breast cancer	Increased CYP2C19 -Increased EET levels -Increased cell proliferation -Metastasis Linked to FABP4/5, PPAY-γ, and SREBP-2	(Apaya et al., 2020)
CYP2J2	Major epoxygenase	Lung cancer Breast cancer Prostate cancer Renal cancer Colorectal cancer Ovarian cancer Bladder cancer	Increased CYP2J2 expression. -Increased EET levels -Increased tumor grade -Increased tumor size Potential therapy: dual-action sEH and COX-2 inhibitors showed increasing level of EETs and suppression of tumor growth in NDL/FVB mouse model.	(Jiang et al., 2005; Jiang et al., 2007; Freedman et al., 2007; Jiang et al., 2009; Chen et al., 2012; Wei et al., 2014)
CYP3A5	Hydroxylase	Prostate cancer (adjacent normal tissue)	Increased production of 20-HETE in adjacent normal tissue -Induces growth and proliferation of tumor -Possibly through GPR75 pathway	(Leskelä et al., 2007; Garcia et al., 2017; Cárdenas et al., 2019)
CYP4A11	ω-Hydroxylases	Lung cancer Breast cancer	-Increased tumor growth -Increased tumor proliferation	(Leclerc et al., 2010; Yu et al., 2011)
CYP4F2	ω-Hydroxylases	Lung cancer Breast cancer Ovarian cancer	Increased CYP4F2 expression -Increased 20-HETE -Pro-inflammatory	(Leclerc et al., 2010; Alexanian et al., 2012)
CYP4A22 CYP4F3 CYP4F11 CYP4F12	ω-Hydroxylases	Lung cancer	Unknown	(Leclerc et al., 2010; Alexanian et al., 2012; Alexanian and Sorokin, 2013)
CYP4Z1	Epoxygenase	Breast cancer	-Pro-angiogenic	(Yu et al., 2012; McDonald et al., 2017)

### Lung Cancer

In 2020, it is estimated that approximately 1,806,590 new cancer cases will be diagnosed in the United States. Of those, 228,820 cases of lung cancer (116,300 in men and 112,520 in women) will be diagnosed, representing roughly 13% of all new cancer diagnoses among adults in the United States (Siegel et al., 2020). Cigarette smoking is the most common, and major, known risk factor for lung cancer where epidemiologic trends strongly correlate with smoking patterns (Mao et al., 2016; Nasim et al., 2019). In the U.S. and most Western countries, the epidemic of cigarette smoking related lung cancer has declined steadily in recent years due to regulations and implementation of smoking cessation programs (Peto et al., 2000). However, in countries such as China, where smoking prevalence has yet to peak, significant increases in lung cancer cases will continue for the foreseeable future (Alberg et al., 2005). Other risk factors include exposure to environmental toxins that could potentially be bioactivated by CYPs to reactive intermediates affecting the lung (Hukkanen et al., 2002). Despite the declining trend in certain parts of the globe, lung cancer remains one of the leading causes of cancer related deaths worldwide and will be responsible for approximately 25% of all cancer related deaths in the U.S. in 2020 (Mao et al., 2016; Siegel et al., 2020).

Non-small cell lung cancer (NSCLC) and small cell lung cancer (SCLC) are two main types of lung cancer representing approximately 80% and 20% of all diagnoses, respectively. (Zheng, 2016). Several studies revealed that duration and number of cigarettes smoked significantly increased the likelihood of squamous cell carcinoma, a sub-type of NSCLC, and SCLC (Auerbach et al., 1975; Morabia and Wynder, 1991; Sobue et al., 2002). The treatment regimen for NSCLC generally includes surgical resection followed by adjuvant therapy whereas the more aggressive SCLC treatments rely exclusively on chemotherapy and targeted immunotherapy (Zhao et al., 2018; Duma et al., 2019). Despite best efforts, mortality rates remain among the highest compared to other cancers. This is mainly because nearly 70% of patients have metastatic disease by the time of diagnosis. Lung cancer is inherently resistant to chemotherapeutics while safe resection of the tumor is often difficult and associated with a high propensity for recurrence (Molina et al., 2008; Leclerc et al., 2010).

**Figure 1:**
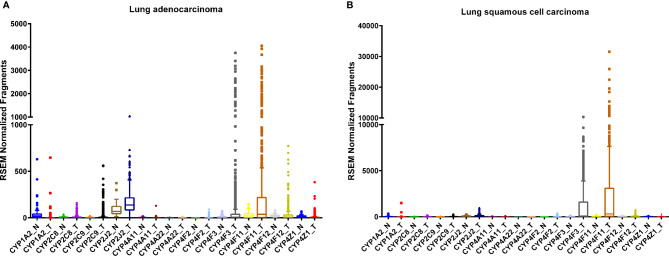
RNA sequencing data obtained from the TCGA on the CYP expression in lung tumor (T) tissues and adjacent normal (N) tissue. Lung adenocarcinoma, **(A)** normal (n = 53) and tumor (n = 465); tissues and squamous cell carcinoma **(B)**, normal (n = 45) and tumor (n = 452) tissues.

The human lung consists of a diverse array of cell types, each with a unique expression pattern of CYPs, many of which are involved in AA metabolism (**Figure 1**). As a member of the CYP1 family, CYP1A2 is expressed in the lung (**Table 1**) and is highly induced by tobacco smoke (Zevin and Benowitz, 1999; Wei et al., 2001; Ding and Kaminsky, 2003) although there are conflicting reports on the level of basal expression of CYP1A2 in the lung (Shimada et al., 1996; Leclerc et al., 2010). CYP1A2 plays a key role in bioactivating inhaled environmental or tobacco smoke procarcinogens increasing susceptibility to lung cancer (Pavanello et al., 2012). In addition to xenobiotic metabolism, CYP1A2 oxidizes AA mainly to 8,9-EET (Rifkind et al., 1995). Currently, there are very few studies elucidating the role of CYP1A2 as an AA epoxygenase in cancer development or progression, however, it remains possible that the formation of the pro-angiogenic EETs by CYP1A2 can play a role in lung cancer progression.

CYP2J2 is another epoxygenase expressed in human lung (**Table 1**) (Zeldin et al., 1996a). It is known to generate all four regioisomers of *cis*-EETs, 14,15-, 11,12-, 8,9-, and 5,6-EET, and no hydroxylation products (Wu et al., 1996; Aliwarga et al., 2017). Jiang et al. reported that CYP2J2 mRNA and protein levels are upregulated, to varying degrees, in eight different tumor derived cell lines, including A549 from lung cancer, compared to non-cancerous cells (Jiang et al., 2005). When CYP2J2 is overexpressed in Tca8113 adenocarcinoma derived cells using an adeno-associated virus, apoptosis was attenuated, and cell migration was enhanced. Similar results were obtained when the Tca8113 cells were treated with EETs while silencing of *CYP2J2* expression resulted in the opposite effect implicating CYP2J2 and EETs in promoting a neoplastic phenotype. (Jiang et al., 2005). Findings from the same study show increases in CYP2J2 mRNA and protein levels in lung squamous cell carcinoma, lung adenocarcinoma, and small cell lung carcinoma tissues relative to adjacent normal tissue analyzed by RT-PCR, Western blotting, and immunohistochemistry, respectively. (Jiang et al., 2005).

CYP2J2 increased lung metastasis in mice xenograft models, independent of primary tumor size, (Jiang et al., 2007) while chemically inhibiting CYP2J2 activity, using compounds structurally related to terfenadine, significantly reduced the tumor cells' ability for adhesion and invasion (Chen et al., 2009). Another study demonstrated that reducing CYP2J2 protein by increasing miRNA let-7b levels resulted in reduced cell growth, increased apoptosis, and metastasis (Chen et al., 2012). Taken together, there is strong evidence that CYP2J2 alters the progression of lung cancer through EET formation and targeting CYP2J2 activity may be a useful therapeutic strategy.

CYP2C9 is also observed in the human lung (**Table 1**) and predominately forms 14,15-EET and 11,12-EET. (Rifkind et al., 1995; Shimada et al., 1996; Hukkanen et al., 2002). An increase in endothelial cell proliferation was observed in HUVEC cells overexpressing CYP2C9 compared to cells with CYP2C9 silenced or treated with sulfaphenazole, a specific CYP2C9 inhibitor (Michaelis et al., 2003). CYP2C9 is highly polymorphic with nearly 33 identified genetic variants (Wang et al., 2009). Compared to the wildtype CYP2C9*1, two extensively studied allelic variants CYP2C9*2 and CYP2C9*3 are known to have significantly reduced enzymatic activity (Zhou et al., 2010). Sausville et al. reported that mice injected with human NSCLC cells expressing CYP2C9*2 and *3 variants produced significantly lower EETs resulting in smaller and less vascularized tumors compared with mice injected with human NSCLC cells expressing wildtype CYP2C9*1 (Sausville et al., 2018). This study shows that CYP2C9 allelic variants with reduced enzymatic activity favorably alter susceptibility to lung cancer.

CYP4A and CYP4F sub-families are major ω-hydroxylases responsible for converting AA to several HETE regioisomers including 19- and 20-HETE (Powell et al., 1998; Gross et al., 2005; Kroetz and Xu, 2005). The expression of CYP4A and 4F families in the human lung has not been extensively studied and therefore, currently, the data are sparse. Trace levels of CYP4A11, CYP4A22, and CYP4F2 mRNA were detected in normal human lung tissue while low-to-moderate mRNA levels were detected for CYP4F3, CYP4F11, and CYP4F12 by RT-PCR (Leclerc et al., 2010). In a study comparing mRNA levels of major ω-hydroxylases in human cancers, upregulation of CYP4A and CYP4F enzymes in NSCLC tissue samples compared to matched normal tissue was observed (Alexanian et al., 2012; Alexanian and Sorokin, 2013). Overexpression of CYP4A11 in a NSCLC derived A549 cell line also increased 20-HETE production and significantly induced cell invasion (Yu et al., 2011). In a murine xenograft model, tumor size and angiogenesis were significantly potentiated in mice overexpressing CYP4A11 through upregulation of VEGF-A and matrix metallopeptidase-9 (MMP-9). Similar effects were observed with treatment of WIT003, a stable 20-HETE analog, and mitigated by administration of HET0016, a potent inhibitor of 20-HETE biosynthesis (Yu et al., 2011). Additionally, CYP4A expression in tumor-associated macrophages promotes pre-metastatic niche formation in lung which is attenuated in the presence of HET0016 strongly supporting the role of CYP4A and 20-HETE in cancer metastasis (Chen et al., 2017).

### Breast Cancer

**Figure 2:**
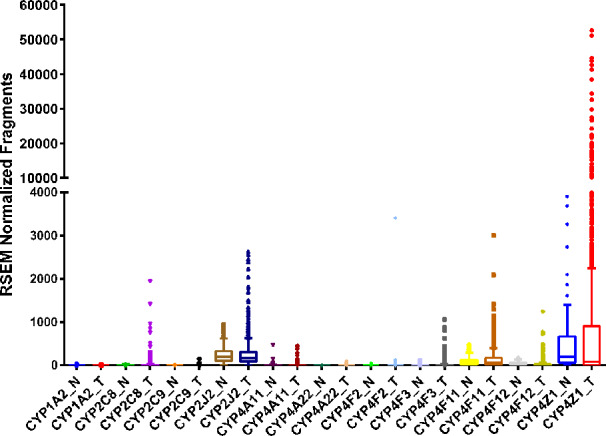
RNA sequencing data obtained from the TCGA on the CYP expression in breast invasive adenocarcinoma tumor (T, n = 992) tissue and adjacent normal (N, n = 101) tissue.

Breast cancer is one of the most prevalent cancers in women, with greater than 270,000 new cases and over 40,000 deaths predicted in the United States in 2020 (Siegel et al., 2020). Numerous studies have examined the expression of several CYP isoforms in breast cancer, including aromatase/CYP19A1 (Suzuki et al., 2009), CYP1A1 (Androutsopoulos et al., 2009; Rodriguez and Potter, 2013), CYP1B1 (McKay et al., 1995; Murray et al., 1997), CYP2J2 (Jiang et al., 2009; Murray et al., 2010), CYP2C (Huang et al., 1996; Murray et al., 2010), CYP3A (Huang et al., 1996), and the CYP4 family (Rieger et al., 2004; Alexanian et al., 2012). Differences in CYP expression in tumor tissue compared to adjacent normal tissue have also been catalogued in several repositories, such as the TCGA database (**Figure 2**).

All the CYPs mentioned above, apart from aromatase, can oxidize AA. CYP1A1 oxidizes AA to 19-HETE (Choudhary et al., 2004), 11,12- and 14,15-EET (Choudhary et al., 2004; Fer et al., 2008) as major products. Likewise, CYP1B1 also mediates both HETE and EET production (Choudhary et al., 2004), with preference for mid-chain HETEs with terminal HETEs and EETs production being relatively equal (Choudhary et al., 2004). CYP2J2, as mentioned above, is exclusively an epoxygenase expressed mostly in the heart, intestines, kidneys, and other tissues (Wu et al., 1996; Zeldin et al., 1997; Michaud et al., 2010; Xu et al., 2013) with increased, robust expression in multiple cancer cell lines and tissues including breast cancer (Jiang et al., 2005; Apaya et al., 2019). The CYP2C enzymes are also primarily AA-epoxygenases (Fer et al., 2008) but have been shown to act as hydroxylases as well (Rifkind et al., 1995). In contrast, CYP3A4/5 are primarily hydroxylases but epoxygenase activity has also been reported (Rifkind et al., 1995; Fer et al., 2008). Lastly, as previously mentioned, the CYP4 family are exclusively terminal chain hydroxylases.

In recent years, research has focused on the effects of eicosanoids on the progression of breast cancer tumors. Elevated EET levels in patients are linked to higher CYP2C and CYP2J2 mRNA and protein expression in tumor cells compared to adjacent normal cells (Wei et al., 2014; Apaya et al., 2019). In addition to elevated CYP epoxygenase expression, studies show reduced levels of soluble epoxide hydrolase (sEH), the enzyme responsible for the hydrolysis of EETs to the dihydroxyeicosatrienoic acids (DHETs), depicted in **Scheme 1** (Wei et al., 2014). The result is increased EET biosynthesis and reduced metabolism, and overall increase in tumor EET levels. Interestingly, Wei's study showed that the CYP2C enzymes were correlated with Ki67 status, a measure of the number of cells that are actively dividing, while CYP2J2 levels were correlated with tumor grade and size (Wei et al., 2014). This may indicate that CYP2C and CYP2J2 regulate distinct pathways necessary for cell proliferation and migration, summarized in **Table 1**. EETs have been shown to not only promote cell survival and growth but also promote angiogenesis, a process critical to sustain tumor growth.

Another study examining the effect of 14,15-EET in breast cancer demonstrated that 14,15-EET mediates epithelial-mesenchymal transition (EMT) in breast cancer cell lines (Luo et al., 2018). EMT of cells is indicative of their metastatic capability as cells become more migratory and invasive. Luo et al. identified FAK/PI3K/AKT activation in response to 14,15-EET, through the upregulation of αvβ3 integrin, with the responses attenuated by 14,15-EEZE, a 14,15-EET antagonist. Lastly, the investigators observed that in addition to promoting mesenchymal properties, 14,15-EET also protected breast cancer cells from cisplatin toxicity *in vitro* and *in vivo* using a mouse xenograft model (Luo et al., 2018). This is significant because it provides evidence of a novel role for 14,15-EET in cancer progression. The other EETs (11,12- 8,9- and 5,6-EETs) were not investigated, and thus it is unclear if they also promote similar resistance to chemotherapeutic intervention. Unfortunately, Luo et al. specifically examined 14,15-EET and did not identify any specific epoxygenase responsible for its biosynthesis although CYP4Z1 is a possible candidate (McDonald et al., 2017). Studies by Luo et al. and Wei et al. both focused primarily on 14,15-EET and while the four isoforms of EETs are typically thought to elicit similar responses, there are no studies that identify definitive and distinct pathways for each regioisomer, if they exist. It is also important to note that different studies disagree on the ratios of EETs formed from individual CYP isoforms (Wu et al., 1996; Imaoka et al., 2005; Fer et al., 2008). Therefore, it is possible that specific EET regioisomers impact distinct aspects of breast cancer, from cell growth and division to overall tumor size, which may partially explain differences in correlation between CYP isoforms and either fraction of dividing cells or tumor grade and size. Nevertheless, the link between CYP epoxygenases, EETs and breast cancer growth and progression led many groups to propose CYP epoxygenases and sEH as potential therapeutic targets for new treatments (Jiang et al., 2007; Wei et al., 2014; Apaya et al., 2019).

A recent study by Apaya et al. examined if CYP2C19 and EET metabolites can be targeted to reduce breast cancer proliferation and prevent metastasis (Apaya et al., 2020). Their study demonstrated that elevated CYP2C19 leads to increased EET levels in a highly metastatic breast cancer cell line, LM6, and that using short hairpin RNA (shRNA) to silence *CYP2C19* expression reduces EET levels and decreases the cell's metastatic potential. Addition of external EETs negated the effects of *CYP2C19* silencing, indicating that the cells' invasive phenotype is, in part, EET-driven. The investigators also linked the pro-metastatic effects of EETs to fatty acid binding proteins (FABP), specifically FABP4 and FABP5, activating PPAR-γ and SREBP-2, resulting in downstream pro-oncogenic gene activation. Downregulation of FABP4/5 attenuated EET activation of pro-metastatic characteristics like cell migration, motility, invasiveness, and colony formation of cells *in vitro* and tumor growth and metastasis in an *in vivo* mouse xenograft model. Apaya et al.'s study provides important insight into the mechanistic cascade involved in EET-mediated metastasis in breast cancer. While their work focused solely on CYP2C19, it would be of interest to determine if inhibiting other CYP epoxygenases yield similar results.

CYP hydroxylases and 20-HETE have also been reported to affect breast cancer pathophysiology. Alexanian et al. demonstrated increased CYP4 enzyme levels in breast cancer tissue compared to normal mammary tissue (Alexanian et al., 2012). The CYP4 family are the primary source of 20-HETE, particularly CYP4F2 and CYP4A11. Borin et al. demonstrated that HET0016 decreases tumor growth and proliferation in a mouse xenograft model through reduction of angiogenic growth factors (Borin et al., 2014), indirectly suggesting that CYP4 enzymes, and other CYP hydroxylases, may be therapeutic targets through inhibition of AA metabolism to 20-HETE.

In a more recent study, Borin et al. showed that HET0016 also decreases tumor growth and metastasis *in vivo* (Borin et al., 2017), implying additional roles for CYP4 enzymes and their 20-HETE product in breast cancer metastasis. There were decreases observed in PI3K/AKT signaling and reduced levels of MMP2 and MMP9, proteins involved in the metastatic process, in mice treated with HET0016. In both their studies, while the investigators attributed the observed effects to inhibition of 20-HETE formation, they fell short of identifying the CYPs involved in their study nor did they measure changes in 20-HETE levels pre and post HET0016 treatment. It remains ambiguous whether the effects observed are due to inhibition of a CYP ω-hydroxylase or some off-target effect of HET0016. Further studies are needed in order to definitively link HET0016's anti-tumor and anti-metastatic effects to 20-HETE and identify the CYP ω-hydroxylases involved.

CYP4Z1 is an interesting isoform overexpressed in breast cancer. Despite being part of the CYP4 family, CYP4Z1 has recently been identified as an AA epoxygenase rather than a hydroxylase (McDonald et al., 2017), exclusively forming 14,15-EET. Previously, Yu et al. linked this enzyme to poor prognosis and worse tumor grades (Yu et al., 2012). The investigators demonstrated that CYP4Z1 promotes overexpression of the pro-angiogenic factor VEGF-A and the suppression of TIMP-2 (a tumor suppressor) in breast cancer cell lines. In *in vivo* models, the investigators also showed CYP4Z1 overexpression resulted in increased tumor size, weight, and vascularization, indicating a role for CYP4Z1 in breast cancer tumor growth and progression. These effects were surprisingly reversed by HET0016 (Yu et al., 2012). These results are puzzling since McDonald et al. convincingly demonstrated that CYP4Z1 lacks AA hydroxylase activity, instead producing only the 14,15-EET regioisomer using *S. cerevisiae* expressed human CYP4Z1. Unfortunately, Yu et al. did not examine directly which eicosanoid was responsible for the effects observed with CYP4Z1 overexpression. Further studies are therefore required to delineate the role CYP4Z1 plays in breast cancer, specifically identifying the metabolite responsible for inducing growth and angiogenesis, as well as how HET0016 is able to mitigate the effects of CYP4Z1 overexpression.

### Prostate Cancer

The American Cancer Society estimates that the prostate will be the leading site of new cancer cases and the second leading cause of cancer deaths among men in the U.S. in 2020 (Siegel et al., 2020). Several studies have examined CYP expression in prostate tissue, both normal and cancerous. Like breast cancer, prostate cancer is sensitive to hormone signaling, specifically by androgens. Therefore, many early studies focused on CYPs known to play a role in hormone metabolism. For example, a study by Finnstrom et al. (2001) investigated gene expression of a panel of drug metabolizing CYP isoforms in normal and cancer prostate tissue in 24 patients. The study is limited in its sample size and focused only on gene expression. CYP1B1 and CYP3A5 were predominant in this sample, both of which have been shown to metabolize hormones and known carcinogens. It should be noted that CYP3A5, as well as CYP3A4, are known AA hydroxylases (Rifkind et al., 1995). Additionally, Choudhary et al. demonstrated that CYP1B1 metabolizes AA, though with less specificity than the CYP3A isoforms, forming internal and terminal HETEs and EETs (Choudhary et al., 2004). A separate study observed CYP2C8, CYP2C9, and CYP2J2 mRNA and protein expression in prostate cancer derived cell lines (Nithipatikom et al., 2010). While this is of interest, studies demonstrating an overexpression of these isoforms in prostate cancer tissue compared to matched normal tissues are needed. Nevertheless, the presence of CYP epoxygenases in prostate cancer models suggests that CYP derived eicosanoids may be involved in prostate cancer progression.

Mid-chain HETEs are typically produced from AA by enzymes in the LOX pathway. Choudhary et al.'s findings, however, indicate that not only is CYP1B1 able to form internal and mid-chain HETEs, they are the preferred metabolites (Choudhary et al., 2004), comprising approximately 50% of metabolites produced. This is of particular interest as studies show that mid-chain HETE serum concentrations are elevated in patients with advanced prostate cancer (Rodríguez-Blanco et al., 2014), indicating that perhaps CYP1B1 and mid-chain HETEs contribute to prostate cancer progression. Rodriguez-Blanco et al.'s study specifically identified 5-, 8-, 11-, and 15-HETE (Rodríguez-Blanco et al., 2014). 5-HETE and 15-HETE, in particular have been shown to induce cell growth and proliferation *in vitro* in various tumor-derived cell lines (O'Flaherty et al., 2005; O'Flaherty et al., 2013; Cabral et al., 2013; Ma et al., 2013), implying a function for these compounds, and thus CYP1B1, in prostate tumor growth and proliferation.

CYP1B1 in prostate cancer was recently linked to caspase-1 activation by Chang et al. (Chang et al., 2017). Caspase-1 is a pro-inflammatory protease that induces programmed cell death (Zhang et al., 2003). The study by Chang et al. demonstrated an inverse relationship between CYP1B1 and caspase-1 expression. Silencing CYP1B1 expression *via* shRNA resulted in increased caspase-1 expression, and attenuated tumor growth and progression in an *in vivo* mouse model (Chang et al., 2017). Unfortunately, the investigators did not measure eicosanoid levels and the exact mechanism by which CYP1B1 regulates caspase-1 expression remains unknown. It is plausible that an eicosanoid product of CYP1B1 suppresses caspase 1 expression and when CYP1B1 is silenced, the eicosanoid levels drop, and caspase-1 expression is activated. It would be of interest in the future to determine if overexpression or silencing of CYP1B1 affects eicosanoid levels in prostate cancer tissue and how individual eicosanoids affect caspase-1 expression and activity *in vitro* and *in vivo*.

In addition to formation of mid-chain HETE metabolites of AA, CYP1B1 is also capable of generating both terminal HETE and EET metabolites, which make up the other 50% of CYP1B1 products (Choudhary et al., 2004). It is therefore possible that CYP1B1 contributes to tumor progression through similar mechanisms as CYP3A5 by producing terminal HETE products that act as autocrine signaling molecules by activating the 20-HETE/GPR75 signaling cascades previously mentioned. The effect of CYP1B1 on tumor formation and growth may be further exacerbated by the simultaneous production of EETs and mid-chain HETEs. EETs are known potent pro-angiogenic factors. This is important because rapidly growing solid tumors are limited by lack of new blood vessels and require angiogenesis to produce new vessels to sustain continuous growth and division (Folkman et al., 1971). The production of EETs within the tumor bed provides a mechanism for tumor cells to stimulate angiogenesis and a means to overcome this growth-limiting problem.

A study using immunohistochemistry observed CYP3A5 expression to be in adjacent non-tumor cells and not in the tumor itself (Leskelä et al., 2007). This is indicative of paracrine signaling that supports tumor growth, progression, and metastasis, where adjacent non-tumor cells produce 20-HETE with detrimental effects on tumor cells. A series of recent studies have identified some key roles for 20-HETE in prostate cancer progression. Garcia et al. first identified GPR75 as a receptor for 20-HETE (Garcia et al., 2017) and Cardenas et al. demonstrated that the 20-HETE/GPR75 signaling pathways contributed to metastatic prostate cancer progression (Cárdenas et al., 2019). Upon exposure to 20-HETE, the prostate cancer cell line PC-3 internalized GPR75, resulted in the phosphorylation of signaling molecules EGFR, NF-κB, and AKT, and altered cellular localization of AKT, NF-κB, and PKCα, all of which were reversed with the addition of GPR75 antagonist (Cárdenas et al., 2019). While several key players have been identified, the full signaling cascade remains to be elucidated. Cardenas et al. also looked at the downstream consequences of PC-3 cell exposure to 20-HETE, demonstrating that the cells exhibit pro-metastatic characteristics (Cárdenas et al., 2019). Their results offer promising insight into how 20-HETE might affect prostate cancer tumor growth and progression. Further studies are needed to determine whether these results can be replicated using animal models or if this is a general mechanism for 20-HETE function in other cell types.

To date, studies examining the direct effects of EETs on prostate cancer progression *in vivo* are absent. However, a study by Vanella et al. demonstrated that ellagic acid, an antioxidant with potential chemotherapeutic properties, may act to inhibit tumor growth by decreasing EET formation, specifically by downregulating CYP2J2 and upregulating soluble epoxide (sEH) expression in a prostate cancer cell line (Vanella et al., 2013). Their study also showed that ellagic acid also acts to decrease CYP4 isoform expression, and by extension, 20-HETE formation. While this study provides some experimental evidence that EETs and 20-HETE have significant roles in prostate cancer as pro-angiogenic factors, it is important to note that the investigators measured mRNA levels of these enzymes, both epoxygenases and hydroxylases, but did not measure protein expression or eicosanoids. Nevertheless, this study marks a starting point for investigating a causal relationship between CYP-mediated AA metabolites and prostate cancer progression.

CYP2C9 expression has also been observed by Enayetallah et al. (Enayetallah et al., 2006) by immunohistochemistry in prostate tumors, however, the study design did not show CYP2C9 expression in adjacent normal tissue, therefore, it is unclear whether CYP2C9 is upregulated in this particular cancer. Regardless, CYP2C9 is an AA epoxygenase, producing all four EET isomers (Fer et al., 2008). As with the other epoxygenases that were previously discussed, CYP2C9 may be involved in tumor growth and progression through EET-mediated angiogenesis, but further studies are needed to provide experimental evidence for such a role.

### Renal Cancer

Kidney cancer is among the top ten diagnosed cancers in the United States for men and women (Siegel et al., 2020). It is estimated that 73,750 adults (45,520 men and 28,230 women) will be diagnosed with kidney cancer in the U.S., of which approximately 85% to 90% of all kidney cancer will be renal cell carcinoma (RCC) (Nabi et al., 2018; Siegel et al., 2020). RCC develops from the proximal epithelial tubules in the renal cortex and is further divided into three histologically distinct subtypes with, the most common, clear cell RCC (ccRCC) representing 70% to 80% of all RCC cases, papillary RCC and chromophobe RCC representing 10% to 15% and 5% of all RCC cases, respectively (Rini et al., 2009; Saad et al., 2019). RCC neoplasms in the collecting duct have also been identified but these tumors are very rare (Sanchez and Simon, 2018; Capitanio et al., 2019; Saad et al., 2019). Among all subtypes of RCC, ccRCC is the most aggressive, and while prognosis for early stage disease is usually promising, advanced stage ccRCC is the most lethal urological malignancy (Rini et al., 2009; Saad et al., 2019).

The proximal tubules of the human kidney express drug metabolizing enzymes as well several CYPs involved in AA oxidation (Shah et al., 2017). Expression of CYP2C8, CYP2C9, and CYP2J2 in human kidney is contentious at best (Knights et al., 2013). Studies by Zeldin et al. did not detect CYP2C8 mRNA transcripts or protein in kidney microsomal fractions by Northern or western blot analyses, respectively (Zeldin et al., 1996b; Lasker et al., 2000). However, Enayetallah et al. reported that CYP2C8 is detected in one of the two normal kidney tissues they analyzed by immunohistochemistry (Enayetallah et al., 2006). Baker et al. reported inconclusive results of CYP2C9 in kidney due to very low abundance bands observed on the western blot suggesting that CYP2C9 is not robustly expressed in kidney. (Baker et al., 2001). In the same study by Enayetallah et al. above, positive immunohistochemical staining for CYP2C9 was observed in the two normal kidney tissue samples analyzed (Enayetallah et al., 2006). And finally, Wu et al. reported very low basal expression of CYP2J2 mRNA in the kidney (Wu et al., 1996).

Enayetallah et al. also examined the protein expression of three CYP epoxygenases in RCC tumor tissue, surrounding non-neoplastic tissue, and benign control tissue by immunohistochemistry. They observed that the staining intensities for CYP2C8, CYP2C9, and CYP2J2 decreased in RCC tissue and surrounding non-neoplastic tissue compared to normal control tissue suggesting a seminal role in carcinogenesis (Enayetallah et al., 2006). In the same study, an anomaly was observed in one of the ten RCC tissues analyzed where there was a significant increase in CYP2J2 staining intensity, relative to the control, and delineates a need for a larger sample size.

**Figure 3:**
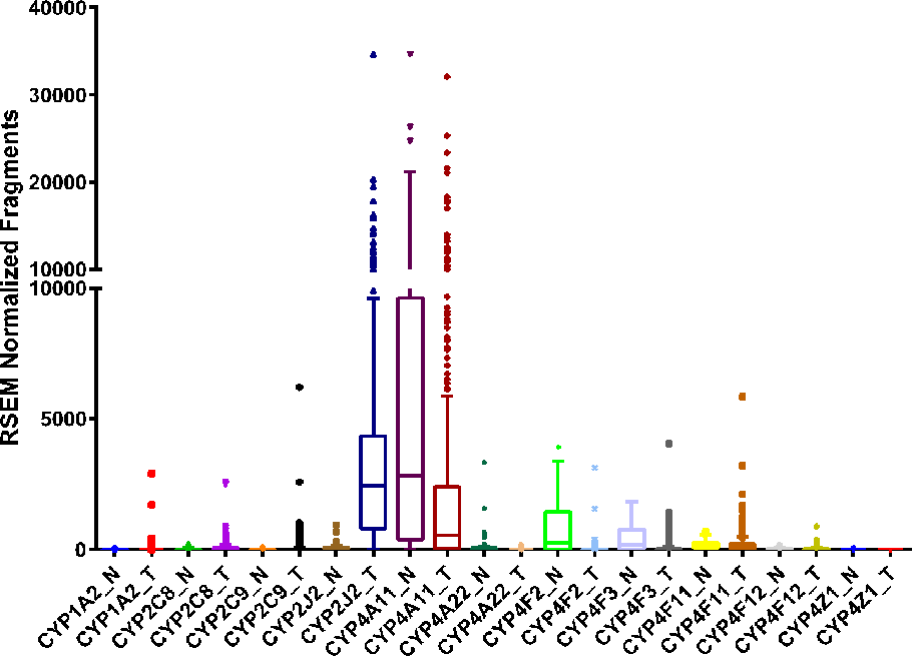
RNA sequencing data obtained from the TCGA on the CYP expression in clear cell renal cell carcinoma tumor (T, n = 480) tissue and adjacent normal (N, n = 64) tissue.

Compared to the CYP2C8 and CYP2C9 RNA sequencing data from The Protein Atlas database, CYP2J2 is highly expressed in renal cancers (https://www.proteinatlas.org/ENSG00000134716-CYP2J2/pathology/renal+cancer). Similarly, RNA sequencing data from the TCGA corroborates the Protein Atlas data and shows CYP2J2 to be overexpressed in ccRCC tumors, while changes in CYP2C8 or CYP2C9 are not significant (**Figure 3**). Overall, the expression of CYP epoxygenases in kidney remains equivocal and future investigations with a large sample size are needed.

EET derived metabolites of AA can promote kidney mesangial cell proliferation inferred from the significantly reduced cell growth following treatment with selective CYP epoxygenase inhibitors (Sellmayer et al., 1991). Yang et al. showed that overexpression of CYP epoxygenases, which in turn increase EET biosynthesis, attenuated TNF-α induced endothelial cell apoptosis (Yang et al., 2007). Further, 14,15-EET protects against serum-withdrawal induced apoptosis in an adherent proximal tubule-like epithelial cell line, LLCPKc14, derived from pig (Chen et al., 2001). The function of CYP epoxygenases, and individual EETs, in RCC has not been extensively studied. However, current studies indicate that EETs, especially 14,15-EET, may have the potential to alter the susceptibility to RCC progression.

CYP4A and CYP4F enzymes responsible for 20-HETE formation are highly expressed in normal renal epithelial cells (Simpson, 1997; Knights et al., 2013). Alexanian et al. showed that renal adenocarcinoma cells maintain the ability to generate 20-HETE through the expression of CYP4F isoforms (Alexanian et al., 2009). Upon inhibition of 20-HETE production with HET0016 and WIT002, cell proliferation is reduced *in vitro* in 786-O and 769-P cells, immortalized RCC derived cell lines, while minimal effects are observed in primary human proximal epithelial cells. Upon implantation of 786-O cells in nude mice, treatment with WIT002 reduced tumor growth by 84% compared to vehicle control (Alexanian et al., 2009). Together these studies indicate inhibition of 20-HETE synthesis by targeting CYP4s could be protective and reduce tumor growth in RCC.

### Colorectal Cancer

The American Cancer Society estimated that there are approximately 147,950 new cases and 53,200 deaths associated with colorectal cancer in the United States in 2020 (Siegel et al., 2020). Although mortality rate has been gradually declining in the United States, colorectal cancer remains the second leading cause of cancer deaths worldwide (World Health Organization, 2018; Siegel et al., 2020). Depending on where the cancer originates, it is called colon or rectal cancer. However, due to the many common features between these cancers, they are often grouped together and referred to as colorectal cancer. Age, lifestyle, genetics, environmental, and dietary factors have been implicated in the etiology of colorectal cancer (Global Burden of Disease Cancer Collaboration et al., 2017; Siegel et al., 2020).

Most studies on eicosanoids involved in the growth and progression of colorectal cancer examined AA metabolites from the COX and LOX pathways. However, a handful of studies investigated epoxygenases and their metabolites. In an earlier study, both protein and transcript of CYP2J2 were observed in LS-174 cell line derived from human colon cancer (Jiang et al., 2005). Although mRNA transcripts of CYP epoxygenases (CYP2Cs and CYP2J2) in colorectal adenocarcinoma patients in TGCA database did not change compared with controls, transcripts of CYP epoxygenases and protein expression of CYP2C9 were significantly increased in primary human colorectal adenocarcinoma (Wang et al., 2019). In the same study, the investigators used azoxymethane/dextran sodium sulfate (AOM/DSS)-induced colon cancer mice to elucidate EETs involvement in colon tumorigenesis. EET levels in plasma and colon of AOM/DSS-induced colon cancer mice were significantly higher than their corresponding control untreated group. In addition, number of tumors and tumor size, expression of several pro-inflammatory and pro-tumorigenesis markers, and EET levels were significantly lower in *Cyp2c*^-/-^ knockout mice treated with AOM/DSS to induce colon cancer. The results of Wang et al. contradict an earlier study by Zhang et al. (2013). Zhang et al. reported that homozygous sEH knockout mice exhibited significantly lower incidence of colorectal carcinoma and reduced tumor burden. Because sEH is responsible for EET hydrolysis to DHETs (**Scheme 1**), sEH knockout mice are expected to have higher levels of endogenous EETs which in turn promote pro-angiogenic activities, one would expect the observations reported by Wang et al. (2019) rather than by Zhang et al. (2013). It is possible that the phosphatase activity of sEH (Newman et al., 2003; Morisseau et al., 2012), also knocked down in these mice, is somehow responsible for the lower incidence of colorectal carcinoma and reduced tumor burden.

CYP2S1 is also overexpressed in colorectal cancer (Kumarakulasingham, 2005). Studies on CYP2S1, in general, are scarce. However, in the immunohistochemical studies, overexpression of CYP2S1 is associated with poor prognosis (Kumarakulasingham, 2005). In addition, Alexanian et al. reported that transcripts of CYP4F and CYP4A were significantly increased in two (stage IIA and stage IIIC) human colon cancer tissues (Alexanian et al., 2012). Unfortunately, they did not determine protein levels of either CYP4As, CYP4Fs or levels of 20-HETE in normal and cancerous colon tissues.

Several other hydroxylases, including CYP3A4/5 (McKay et al., 1993), CYP1B1 (Choudhary et al., 2004), and CYP2U1 (Chuang et al., 2004), were elevated relative to normal colon tissues (Kumarakulasingham, 2005). Because many of the cancer drugs are cleared by CYP3A, the presence of CYP3A could affect tumor sensitivity to treatments (Martínez et al., 2002). A study using human colorectal adenocarcinoma cell lines HCT116 and SW480 suggested that elevated interleukin-6 downregulated microRNA27b *via* DNA methylation which in turn upregulated the expression of CYP1B1 (Patel et al., 2014). Because of the overexpression of CYP1B1 in cancer tissue, it has been explored as a potential chemotherapeutic target (Li et al., 2017). No follow up studies, as of yet, reported on the importance of elevated levels of CYP2U1 in colorectal cancer (Kumarakulasingham, 2005).

### Ovarian Cancers

The American Cancer Society estimated that there are approximately 21,750 new cases and 13,940 deaths associated with ovarian cancer in the U.S. in 2020 (Siegel et al., 2020). Age, history of breast or colorectal cancer, genetic, endometriosis, and nulliparity have been reported as potential risk factors for ovarian cancer (Kerlikowske et al., 1992; Li and Karlan, 2001; Risch et al., 2001; Watson et al., 2001; Boyd, 2003; Gates et al., 2010; Bonadona et al., 2011; Stewart et al., 2013; Wentzensen et al., 2016).

Differential gene analysis tumor-free specimens from malignant peritoneum and benign peritoneum from patients with benign pelvic disease and tumor specimens obtained from patients with advanced epithelial ovarian cancer showed a significant elevation in the expression of CYP2J2, but not CYP2C8 or CYP2C9, in tumors from patients with advanced epithelial ovarian cancer (Freedman et al., 2007). In addition, an immunohistochemistry study revealed that a minor and understudied CYP epoxygenase, CYP2S1, was elevated in metastatic ovarian cancer which correlated with poor prognosis (Downie et al., 2005). The significance of this finding is yet to be determined.

In a nested case–control study within the screening arm of the Prostate, Lung, Colorectal, and Ovarian Cancer Screening Trial, designed and sponsored by National Cancer Institute, EETs were not associated with increased risk of ovarian cancer (Hada et al., 2019). This does not agree with previous reports that linked EETs to tumor growth and metastasis (Panigrahy et al., 2012; Skrypnyk et al., 2014). Since EETs are known as anti-inflammatory mediators (Node et al., 1999; Campbell, 2000), one would expect they will reduce inflammation that is widely associated with tumorigenesis and cancer progression (Multhoff et al., 2011; Greten and Grivennikov, 2019) thereby reducing the risk of developing ovarian cancer. Reducing inflammation proved protective in a population based, case-control study of women who previously had ovarian cancer and used aspirin and non-steroidal anti-inflammatory drugs continuously. In this population, risk of ovarian cancer was significantly reduced (Lo-Ciganic et al., 2012).

In a study using an array of human cancer cell lines, a combination of low-dose soluble epoxide hydrolase inhibitor and low-dose COX-2 inhibitor did not significantly inhibit cell proliferation (Zhang et al., 2014). In the same study, administration of the dual inhibitor to an *neu* deletion (NDL: Her2^+^, Ki67^+^, ER/PR negative) breast cancer Friend Virus B (FVB) female mouse model demonstrated elevated EETs while suppressing tumor growth (Zhang et al., 2014). Because of the discrepancy between in vitro and in vivo tumor models and the lack of EET levels measurement in the in vitro tumor model, functional characterization of EETs with respect to the anti-inflammatory and angiogenic properties need to be further explored in ovarian cancer.

Expression of several CYP hydroxylases, including CYP1B1 (Choudhary et al., 2004), CYP2U1 (Chuang et al., 2004), and CYP3A (Rifkind et al., 1995; Fer et al., 2008), along with the epoxygenase CYP4Z1 (Downie et al., 2005), were increased in primary ovarian cancer. Nonexpressers of CYP3A5 (carriers of CYP3A5*3) in Chinese epithelial ovarian cancer patients were at an increased risk of developing toxicity induced by the combination of paclitaxel/carboplatin (Hu et al., 2016). In human ovarian cancer tissue, elevated transcript and protein levels of CYP4F2, an ω-hydroxylase, were corroborated with higher arachidonic acid turnover relative to normal tissue (Alexanian et al., 2012). Considering that inflammation is critical in cancer development (Coussens and Werb, 2002) and the pro-inflammatory properties of 20-HETE and mid-chain HETEs (Cole et al., 2013; Hoopes et al., 2015; Powell and Rokach, 2015), elevated HETE levels in ovarian cancer could lead to poor prognosis and increase tumor progression.

### Pancreatic Cancer

The American Cancer Society estimates that over 57,000 new cases of pancreatic cancer will be diagnosed in the United States, with approximately 47,000 deaths due to this cancer in 2020 (Siegel et al., 2020). Few studies examined changes in CYP expression levels in pancreatic cancer. To date, no studies have been conducted to assess the effect, if any, of CYP derived eicosanoids in the development and progression of pancreatic tumors. A single immunohistochemical study by Standop et al. (2003) demonstrated robust protein expression of CYP 1A1, 1A2, 2B6, 2C, 2D6, 2E1, and 3A4, as well as cytochrome P450 oxidoreductase (CPR) in normal pancreatic tissues from 24 individuals sourced from organ donors or autopsy. The investigators were also able to detect increased, though not statistically significant, expression of CYP 1A1, 2B6, 2C, 2D6, 2E1, 3A4 and CPR in pancreatic tumor tissues from 21 donors obtained from surgeries, indicating potential roles for these enzymes in cancer pathogenesis (Standop et al., 2003). There are several limitations associated with this study that need to be considered, primarily, it is unclear whether the increase in CYP expression was due to the cancer phenotype or to other covariates not considered in their study population (such as diabetic status, etc.). Normal and tumor tissue samples were not matched so there is no indication whether these enzymes increased in cancerous tissue relative to healthy tissue in the same patient. Finally, eicosanoid levels were not measured, and there is no indication that elevated CYPs correspond to elevated eicosanoids in pancreatic tissue. As with the other previously described solid tumors, it is likely that any CYP mediated eicosanoid involvement in pancreatic cancer would be due to their anti-apoptotic and pro-angiogenic properties. Currently, however, there is no evidence to suggest that CYP derived eicosanoids are involved in the initiation or progression of pancreatic cancer.

### Bladder Cancer

Bladder cancer is the fourth most common cancer in men and significantly less common in women. It is estimated that 81,400 new cases (62,100 men and 19,300 women) of bladder cancer will be diagnosed in the United States in 2020 (Siegel et al., 2020). Several risk factors have been linked to bladder cancer with the most common bring cigarette smoking. Chemicals found in cigarettes, most notably arylamines including 2-naphthylamine and 4-aminobiphenyl (ABP), increase the susceptibility to bladder cancer by 2- to 4-fold and nearly half of all bladder cancer diagnosed is caused by cigarette smoking (Kirkali et al., 2005). Occupational exposure to these chemicals has traditionally put workers of the textile dye or rubber tire industry at a significantly higher risk for bladder cancer (Melick et al., 1955; Melick et al., 1971; You et al., 1990). CYP1A2 activity is associated with increased risk of bladder cancer due to its role in bioactivating arylamines in cigarette smoke but the role of CYP1A2 epoxygenase activity has not been studied (Lee et al., 1994; Tao et al., 2012). Enayetallah et al. reported expression of CYP2J2, CYP2C8, and CYP2C9 in urothelial cancer indicating a potential role for EETs in urinary tumorigenesis (Enayetallah et al., 2006). Wang et al. reported that CYP2J2 overexpression in human breast cancer cell line promoted metastasis in not only the lungs but also the liver and bladder as well (Panigrahy et al., 2011). However, there are very few studies investigating CYP mediated AA metabolites in bladder cancer or how these metabolites alter disease progression.

## Summary

Many eicosanoid forming CYPs are expressed in solid tumors, and each tumor has a unique expression profile of these isozymes. In general, CYP expression is usually studied in their drug metabolizing capacity, reducing drug levels in tumors and potential contribution to chemotherapeutic resistance. However, due to the involvement of eicosanoids in cancer progression, CYPs acting as sources of HETEs and EETs and comparing eicosanoid levels in tumor compared to healthy tissue are also of interest. EET levels are further elevated as sEH is normally downregulated in cancerous tissue. Studies have linked elevated CYP levels with higher HETE/EET levels and demonstrated roles for these eicosanoids in tumor formation, growth, and metastasis. These studies highlight the potential of targeting CYP enzymes for novel cancer therapeutics.

In nonpathological settings, EETs and HETEs typically have opposing effects. For example, in the vasculature, EETs are potent vasodilators while 20-HETE is a vasoconstrictor. In cancer, both EETs and HETEs seem to have similar outcomes promoting cell growth and metastases. Studies comparing the effects of EETs vs. 20-HETE under comparable conditions are required to determine which eicosanoid is more potent. Similar studies comparing the potency of each EET isomer are also important to identify if they work through similar or different pathways. Most studies investigate, and establish, associations to a single isomer but studies investigating EETs individually and in combination are required to better understand how CYPs and their AA-derived metabolites are involved in tumor growth and progression. Despite the inhibition of these enzymes as promising mechanism for future therapeutic targets, there are no drugs in the clinic that target these pathways to halt or reverse tumor progression. This is likely due to the important physiological functions CYPs and eicosanoids play in non-cancer settings. Further understanding of the pathways triggered by EETs and 20-HETE in cancer could identify additional avenues for future novel therapeutic intervention.

## Author Contributions

All authors contributed to the conception of this review. EE, CC, and TA wrote the first draft of the manuscript. EE and CC prepared schemes and figures. All authors contributed to manuscript revision, read and approved the submitted version.

## Funding

This work was supported by the National Center for Advancing Translational Sciences grant TL1TR000422, and the National Institutes of General Medical Sciences grant T32GM007750.

## Conflict of Interest

The authors declare that the research was conducted in the absence of any commercial or financial relationships that could be construed as a potential conflict of interest.
